# Tumor promoting effects of circRNA_001287 on renal cell carcinoma through miR-144-targeted CEP55

**DOI:** 10.1186/s13046-020-01744-2

**Published:** 2020-12-01

**Authors:** Jiafu Feng, Yongcan Guo, Yuanmeng Li, Jiawei Zeng, Yaodong Wang, Yuwei Yang, Gang Xie, Qian Feng

**Affiliations:** 1 Department of Clinical Laboratory, Mianyang Central Hospital, No. 12, Changjia Lane, Jingzhong Street, Fucheng District, Sichuan Province 621000 Mianyang, PR China; 2 Clinical Laboratory of Traditional, Chinese Medicine Hospital Affiliated to Southwest Medical University, 646000 Luzhou, Province, PR China; 3 Department of Medical Laboratory, Affiliated Hospital of Southwest Medical University, Sichuan Province 646000 Luzhou, PR China; 4 Department of Urology Surgery, Mianyang Central Hospital, Sichuan Province 621000 Mianyang, PR China; 5 Department of Pathology, Mianyang Central Hospital, Sichuan Province 621000 Mianyang, PR China; 6 College of Medical Technology, Chengdu University of Traditional Chinese Medicine, Sichuan Province 610075 Chengdu, PR China

**Keywords:** circ_001287, miR-144, CEP55, Renal cell carcinoma, Proliferation, Migration, Invasion, Apoptosis

## Abstract

**Background:**

Renal cell carcinoma (RCC) is a common urological cancer. circular RNAs (circRNAs) is involved in the development of various types of cancers. However, the roles and underlying mechanisms of circRNAs in RCC are not fully elucidated. Herein, we aimed to examine the potential effect of circ_001287 on RCC progression.

**Materials and Methods:**

Microarray-based gene expression profiling of RCC was initially employed in order to identify differentially expressed genes. Next, the expression of circ_001287 was examined, and the cell line with the highest circ_001287 expression was selected for subsequent investigation. The interaction among circ_001287, miR-144, and CEP55 was identified by conducting luciferase reporter assay, RNA-pull down, RIP, RT-qPCR and FISH. The effect of circ_001287 on proliferative, invasive and migratory capacities as well as tumorigenicity of transfected cells in mice was examined using gain- and loss-of-function experiments.

**Results:**

circ_001287 and CEP55 were highly expressed while miR-144 was decreased in RCC tissues and cell lines. circ_001287 can up-regulate CEP55 by binding to miR-144, which resulted in increased proliferative, invasive and migratory capacities and tumor growth *in vivo*. In addition, down-regulation of miR-144 was also observed to promote these biological activities.

**Conclusions:**

Overall, these results elucidate a new mechanism for circ_001287 in RCC development and provide a potential therapeutic target for RCC patients.

## Background

Renal cell carcinoma (RCC) ranks the third leading cause of death in humans suffering urological tumors [1]. The incidence rate between male and female is 3: 1 and the prevalence increases along with age and reaches the peak at the age between 60 and 70 [2]. RCC is a disorder that results from the renal epithelium and afflicts more than 90% of cancers in the kidney [3]. Patients suffering localized RCC can be treated with nephrectomy. Approximate one quarter of the patients have relapses in distant sites and the distant metastases often occur in the lymph nodes, lungs, livers, bones and brains [4]. The survival rate for patients with maligant RCC is reported to be extremely low [5]. The von Hippel Lindau (VHL) is a typical tumor suppressor gene, and most RCC have VHL gene abnormalities including VHL gene mutations, methylation and loss of heterozygosity [6]. Besides, hypoxia inducible factor (HIF)-1 was inhibited by VHL, which results in inhibition of growth and invasion and metastasis of renal tumor cells [7]. Recently, circular RNAs (circRNAs) demonstrate as prognostic markers in a variety of diseases and cancers [8].

CircRNAs belong to a class of non-coding RNAs without 5’ end caps or 3’ end poly (A) tails [9]. circRNAs often originate from exon regions (called exon cyclic RNA), but may also from endogenous and intergenic regions [10]. circRNAs have unique properties which include the ability of RNA rolling circle amplification, the potency to rearrange genomic information order, and protection from exonucleases due to RNA folding [11]. circRNAs are also important participants in normal cellular differentiation, tissue homeostasis and disease development [12]. Studies have shown the implication of circRNAs in numerous tumors, including colon cancer, esophageal cancer and glioma as expression of specific circRNAs are strongly linked to clinical characteristics of staging, distant metastasis, age of onset and gender [13]. Bioinformatic analysis has shown that some circRNAs possess multiple binding sites for microRNAs (miRNAs) and thus may function as miRNA sponges [14]. miRNAs are endogenous and approximately 22-bp long RNAs that exert a crucial regulatory role at the posttranscriptional level in both animals and plants via targeting mRNAs for cleavage or translational suppression [15, 16]. It has been reported that miR-144 shows an effect on RCC progression by means of inhibiting its target mTOR expression, and thereby miR-144 might serve as a novel modality for RCC treatment [17]. Interaction between miR-144 and centrosomal protein 55 (CEP55) leading to a negative correlation on their expression has been identified in breast cancer [18]. CEP55 composed of many coiled coil proteins [19]. Recently, it has been reported that CEP55 possesses the ability to promote epithelial-mesenchymal transition (EMT) and can be an effective prognostic marker in RCC [20]. Based on the aforesaid information, we proposed a hypothesis that circ_001287 may function in RCC development via regulating miR-144 and CEP55. Therefore, we attempted to verify the expected role of the circ_001287 in miR-144/CEP55 network in RCC and to identify an effective prognostic marker in RCC.

## Materials and methods

### Ethics statement

Ethics Committee in Mianyang Central Hospital (S2014048, S2018085) and Southwest Medical University approved the protocols involved in the current study. Written agreement was acquired from all the participants before the experiments. Animal experiments were implemented following the ethical standards of animal experiment ratified by Animal Management Committee in Southwest Medical University.

### Microarray-based gene expression profiling

RCC-related circRNA dataset (GSE100186 [normal samples = 4, tumor samples = 4]) and gene expression datasets (GSE100666 [normal samples = 3, tumor samples = 3], GSE15641 [normal samples = 23, tumor samples = 32], GSE53757 [normal samples = 72, tumor samples = 72], and GSE71963 [normal samples = 16, tumor samples = 32]) were attained from the Gene Expression Omnibus database. Differential expression was performed using the Limma package in R language to find differentially expressed circRNAs or genes associated with RCC. The basic information of circRNA was queried from the circBase database available at http://www.circbase.org/. The potential circRNA-bound miRNAs were then predicted using the CircInteractome available at https://circinteractome.nia.nih.gov/index.html and starBase available at http://starbase.sysu.edu.cn/index.php databases. DIANA available at http://diana.imis.athena-innovation.gr/DianaTools/index.php?r=microT_CDS/index, Target Scan available at http://www.targetscan.org/vert_71/, miRDB available at http://www.mirdb.org/ [21], microSearch available at http://www.exiqon.com/microrna-target-prediction, and mirDIP available at http://ophid.utoronto.ca/mirDIP/ [22] were employed for miRNA target gene prediction. Then predicted target genes were subjected to comparison with the differentially expressed genes (DEGs) screened from the RCC expression datasets to filter the DEGs regulated by miRNAs. The Venn online analysis tool available at http://bioinformatics.psb.ugent.be/webtools/Venn/ was adopted in order to compare differences among differentially expressed miRNAs or gene sets in different databases.

### Patient enrollment

Seventy-seven RCC patients were enrolled in the present study, including 57 cases of clear cell RCC, 7 cases of papillary RCC and 13 cases of other types RCC. Tumor and adjacent normal tissues were then surgically obtained from these patients who confirmed as RCC by pathology in Mianyang Central Hospital and Traditional Chinese Medicine Hospital Affiliated to Southwest Medical University from January 2015 to September 2019. The patients, including 45 males and 32 females, were aged 35–70 years (57.87 ± 7.33). Table 1 showed the baseline demographic and clinical characteristics of the patients prior to surgery. None of the patients had received chemoradiotherapy before the surgery. After collection, the tissues were immediately stored at -80 °C for subsequent experimentations.

**Table 1:** Baseline demographic and clinical characteristics of the RCC patients before surgery

	CircRNA_001287 Expression		*χ2*	*p*
	Low expression (*n* = 37)	High expression (*n* = 40)		
Age (years)				
< 60	21	21	0.141	0.708
≥ 60	16	19		
Gender				
Male	20	25	0.565	0.452
Female	17	15		
Pathological grade				
I–II	20	32	5.901	0.015
III–IV	17	8		
Lymph node involvement				
NO-N1	5	23	16.071	0.001
N2-NX	32	17		
Distant Metastasis				
Absent	8	23	5.301	0.021
Present	24	22		
Tumor size				
T1-T2	9	25	11.361	0.001
T3-T4	28	15		
TNM stage				
I-II	6	23	13.951	0.001
III-IV	31	17		
UACR (mg/g)				
< 30	23	19	1.667	0.197
≥ 30	14	21		
eGFR (ml/min/1.73 m^2^)				
≥ 60	22	18	1.610	0.204
< 60	15	22		
Cr (µmol/L)				
< 82.1(F)/97.0(M)	22	17	2.212	0.137
≥ 82.1(F)/97.0(M)	15	23		
CysC (mg/L)				
< 1.09	23	18	2.274	0.132
≥ 1.09	14	22		

Note: *CysC* Cystatin C; *UACR* urinary albumin to creatinine ratio; *SCr* serum creatinine; *eGFR* estimated glomerular filtration rate, eGFR = 78.64 CysC^−0.964^; *TNM* tumor node metastasis. The difference in Sex was compared by chi-square test and for the others by Welch t test

### Cell culture

Human RCC cell lines, such as A-498, CAKI-1, and CAKI-2, were subjected to culture with the help of Dulbecco’s Modified Eagle Medium (DMEM; HyClone, South Logan, UT, USA) encompassing 10% fetal bovine serum (FBS) and 100 U/mL of penicillin-streptomycin solution. RCC cell line 786-O was cultured with the help of RPMI)-1640 (HyClone) medium which encompassing 10% FBS and 100 U/mL penicillin-streptomycin solution. Normal renal tubular epithelial HK-2 cell line was subjected to culture with the help of Keratinocyte Serum-Free medium (Gibco, Gaitherburg, MD, USA) which was supplemented with 10% FBS and 100 U/mL penicillin-streptomycin solution. All cell lines were cultured at controlled temperature of 37 °C in an atmosphere of 5% CO_2_ and were screened by RNA isolation and quantification. The aforesaid cell lines were attained from a company named Procell Life Science & Technology Co., Ltd. (Wuhan, China) (http://www.procell.com.cn/).

### Cell grouping and transfection

The RCC cells 786-O were treated with: small interfering RNA (si)-circ_001287-1, si-circ_001287-2, miR-144 inhibitor, overexpression (oe)-CEP55 (plasmid overexpressing CEP55), or corresponding negative controls (NCs). si-circ_001287, miR-144 inhibitor and oe-CEP55 were attained from a company named RiboBio (Guangzhou, China). The target sequence of si-circ_001287 was 5’-ATGCACCAAAGTGATGATTTA-3’. Cells were subjected to seeding into a plate with 24 wells. When the confluence of cells reached 50%-60%, cells were subjected to transfection by means of Lipofectamine™ (Invitrogen, Car, Cal, USA). The complex was added into cells to be transfected for further culture in the complete medium after 6 to 8 h.

### Fluorescence in situ hybridization (FISH)

FISH was implemented using circ_001287 sequence and specific probe of miR-144. Cy5-labeled probe was specific to circ_001287 and farm-labeled probe was specific to miRNA. The nuclei were then subjected to 4,6-diamino-2-phenyl indole staining. All images were obtained using the Zeiss LSM880 NLO confocal microscopy system (Leica Microsystems, Mannheim, Germany).

### RNA isolation and quantification

At 24 h post transfection, RCC cells 786-O in logarithmic growth phase were collected for total RNA extraction by means of Trizol (15,596,026, Invitrogen). The RNA was reversely transcribed into cDNA as per the manuals of PrimeScript RT reagent Kit (RR047A, Takara, Kyoto, Japan). The primers (Table 2) were designed and then synthesized by a company named Shanghai Sangon Biotech Company (Shanghai, China). Thereafter, Reverse transcription quantitative polymerase chain reaction (RT-qPCR) was implemented with ABI7500 fluorescent quantitative PCR. The expression of circ_001287, miR-144 and CEP55 was measured using 2^−△△Ct^ method [23].

**Table 2:** Primer sequences for RT-qPCR

Gene	Sequence (5’-3’)
circRNA_001287	F: GCACTGAGGAACAGGACACA
	R: CCATGGCTTCCTTCTGTGAT
linear_001287	F: CAGCAGCCAACCTTACCTCT
	R: CATGTCCGCAGTCCTACCAA
miR-144	F: GGCCCTGGCTGGGATATCAT
	R: GGTGCCCGGACTAGTACATC
CEP55	F: TGGAGAAAATTCGAGTCCTTGAG
	R: TTTCAACACCTGCTCCCTCC
GADPH	F: CGGAGTCAACGGATTTGGTCGTAT
	R: AGCCTTCTCCATGGTGGTGAAGAC
U6	F: CGCTTCGGCAGCACATATACTA
	R: CGCTTCACGAATTTGCGTGTCA

Note: *RT-qPCR* reverse transcription quantitative polymerase chain reaction; *F *forward; *R* reverse; *CEP55* centrosomal protein 55; *GADPH* glyceraldehyde-3-phosphate dehydrogenase

### RNA-pull down assay

RCC cells 786-O were subjected to transfection with wild type (wt)-bio-miR-144 and mutant type (mut)-bio-miR-144 labeled with 50 nM biotin. Then 48 h later, the cells were harvested and washed with the help of phosphate buffer saline (PBS). Cells were incubated in lysis buffer (Ambion, Austin, Texas, USA) for duration of 10 min and centrifuged at the condition of 14,000 g to attain the supernatant. Thereafter, protein lysate underwent incubation with M-280 streptavidin beads (S3762, Sigma-Aldrich, St Louis, MO, USA) that had been pre-coated with the help of RNase-free bovine serum albumin and yeast tRNA (TRNABAK-RO, Sigma-Aldrich). Then beads were subjected to incubation at controlled temperature of 4 °C for duration of 3 h and 2 washes with the help of pre-cooled lysis buffer, 3 washes with the help of low-salt buffer and 1 wash with the help of high-salt buffer. The bound RNA was finally purified with the help of Trizol, and circ_001287 expression was measured using RT-qPCR.

### RNA binding protein immunoprecipitation (RIP) assay

RCC cells 786-O underwent lysing with the use of lysis buffer (25 mM Tris-HCl, pH = 7.4; 150 mM NaCl; 0.5% NP-40; 2 mM ethylenediaminetetraacetic acid; 1 mM NaF and 0.5 mM dithiothreol) containing RNasin (Takara) and protease-inhibitor (B14001a, Roche, Indianapolis, IN, USA). The supernatant was collected after lysate was centrifuged at condition of 12,000 g for duration of 30 min. The supernatant were then added with anti-human Argonaut-2 (Ago2) magnetic beads (BMFA-1, Biomarker Technologies CO., LTD, Beijing, China) and cells for control were added with anti-immunoglobulin G (IgG) magnetic beads. After incubation at controlled temperature of 4 °C for duration of 4 h, the beads were subjected to washing with wash buffer (50 mM Tris-HCl; 300 mM NaCl, pH = 7.4; 1 mM MgCl_2_; 0.1% NP-40) for three times. RNA was subjected to extraction from magnetic beads with the help of Trizol, and expression of circ_001287 and miRNA-144 was measured using RT-qPCR.

### Western blot analysis

Cells were lysed in radioimmunoprecipitation assay lysis (P0013B, Beyotime, Shanghai, China) encompassing phenylmethylsulfonyl fluoride. A Bio-Rad DC Protein Assay kit (Ewell Bio-technology CO., LTD, Guangzhou, China) was adopted to quantify the protein. The protein was subjected to separation by means of sodium dodecyl sulfate-polyacrylamide gel electrophoresis and then, which was followed by transferring onto polyvinylidene fluoride membranes. The membrane was immersed in 1 × Tris-buffered saline Tween-20 containing 5% non-fat skim milk at the ambient temperature for duration of 2 h. Next, the membrane underwent overnight incubation at controlled temperature of 4 °C with primary rabbit anti-human antibodies (1: 2000; Abcam Cambridge, UK) to CEP55 (ab170414), proliferating cell nuclear antigen (PCNA) (ab18197), KI67 (ab15580), matrix metalloproteinase 2 (MMP2; ab92536), MMP9 (ab76003), Cyclin D1 (ab92536), Cyclin E (ab33911), Caspase-3 (ab13847), and Caspase-9 (ab32539). The membrane underwent further incubation with secondary antibody IgG (Abcam, ab6721, goat anti-rabbit, 1: 20,000) at the ambient temperature for duration of 1 h, followed by development with the help of enhanced chemiluminescence. The densities of proteins were measured by the Image J software. The experiments were repeated three times for statistical analysis.

### Cell counting kit-8 (CCK-8)

Cell proliferation assays were performed with the CCK-8. First, cells were inoculated in 96-well plates with 2,000 cells/well and 100 µL culture medium. After transfection, 10 µL portion of CCK-8 reagent was added into each well and subjected to culture at controlled temperature of 37 °C for duration of 2 h. The wells with the corresponding amount of medium and CCK-8 reagent in the absence of cells were used as control. The measurement of optical density value of each well at 450 nm was implemented by means of a microplate reader. The value was proportional to the number of cell proliferation in the medium, and the growth curve was drawn.

### Transwell assay

A total of 3 × 10^4^ cells/well were cultured in 250 µL portion of medium without serum, pretreated with lycorine and added into the upper chamber, followed by addition of fresh culture medium containing 10% FBS into the basolateral chamber. Thereafter, cells were subjected to incubation at controlled temperature of 37 °C with 5% CO_2_ for duration of 24 h for migration. Invasion ability was measured using the same procedure except that the additives were coated with 200 mg/mL portion of Matrigel. Subsequent to incubation for duration of 48 h, the migrated and invasive cells in the basolateral chamber were subjected to 0.1% crystal violet staining. Images were captured under a phase-contrast microscope [24].

### Flow cytometry

After 48 h of transfection, the apoptosis of RCC cells 786-O was measured by means of Annexin V-fluorescein isothiocyanate/propidium iodide (FITC/PI) double staining kits (556,547, Solja, Shanghai, China). Initially, 10 × binding buffer was subjected to dilution into 1 × binding buffer using deionized water, and cells underwent centrifugation at the condition of 2000 rpm for duration of 5 min and then collected. Next, the cells were subjected to re-suspension with the help of 1 × PBS, centrifugation at 200 rpm for duration of 5–10 min, and suspension in 1 × binding buffer. With the addition of 5 µL portion of Annexin V-FITC, cells were incubated for 15 min at the ambient temperature in dark, whereupon cells were subjected to incubation with 5 µL portion of PI for duration of 5 min and subjected to a flow cytometer (Cube6, Partec, Germany). FITC was measured when wavelength was 480 nm and 530 nm and measurement of PI was implemented when the wavelength was greater than 575 nm.

The transfected cells were subjected to overnight 70% pre-cooled ethanol fixation at controlled temperature of 4 °C, 2 pre-cooled PBS washes after centrifugation, re-suspension in 100 µL portion of PBS, and addition of RNase (1 mg/mL). The final concentration of cell suspension was 50 ug/mL by adding PI staining solution. The cells were stained at controlled temperature of 4 °C for duration of 40 min in the dark and then washed with PBS. The DNA content of cell cycle was measured at the wavelength of 575 nm and the percentage of cell cycle was calculated.

### Dual-luciferase reporter gene assay

The binding sites of circ_001287, miR-144 and CEP55 were analyzed using the bioinformatics website and the specific sequences encompassing the binding sites were obtained. The full length of circ_001287 and the 3’untranslated region (3’UTR) of CEP55 were subjected to being cloned into pmirGLO (E1330, Promega, Madison, WI, USA) luciferase vector and named as pcirc_001287-wt and pCEP55-wt respectively. The pcirc_001287-mut and pCEP55-mut vectors were constructed respectively. pRL-TK vector (E2241, Promega) expressing renilla luciferase was used as the internal control. Luciferase reporter vector (CRL-1415, American Type Culture Collection, Manassas, VA, USA) was then subjected to co-transfection together with mimic-NC and miR-144 mimic into RCC cells 786-O, respectively. The Dual Luciferase Reporter Gene Assay Kit (GM-040502A, Qcbio, Shanghai, China) was employed in order to measure the fluorescence intensity at 560 nm (firefly relative luciferase units [RLU]) and 465 nm (renilla RLU), and the ratio between firefly RLU and renilla RLU was used to determine the binding strength.

### Immunohistochemistry

The specimens were subjected to 10% formaldehyde fixation and preparation of 4 µm paraffin-embedded sections. The sections were then deparaffinized with xylene, dehydrated using gradient ethanol, incubated at controlled temperature of 37 °C in 3% H_2_O_2_ (Sigma-Aldrich) for duration of 30 min, and boiled in 0.01 M citric acid buffer at controlled temperature of 95 °C for duration of 20 min. Thereafter sections were sealed with goat serum working solution at controlled temperature of 37 °C for duration of 10 min, and underwent incubation with primary antibody rabbit anti-human CEP55 (ab214302, 1: 100, Abcam) at controlled temperature of 4 °C for duration of 12 h. Subsequently, the sections were reacted with corresponding biotin-labeled secondary goat anti-rabbit (ab150077, 1:1 00, Abcam) at the ambient temperature for duration of 10 min and horseradish peroxidase-tagged streptomyces ovalbumin working solution at the ambient temperature for duration of 10 min. The sections were visualized with diaminobesidine and stored in the dark at the ambient temperature for duration of 8 min. Next, the sections were stained with hematoxylin, dehydrated, cleared, sealed, and observed under a light microscope. The number of positive cells was counted by Japanese Nikon image analysis software. Three random fields with same magnification (200 ×) were selected so as to calculate the number of positive cells in each section. The cells with over 25% positive staining and obvious tan particles in the cytoplasm were considered as positive cells and the positive rate = number of positive cells/total number of cells [25].

### Xenotransplantation in nude mice

About 1 × 10^7^ cells were subcutaneously injected into the axilla of athymic female BALB/C nude mice (6–8 weeks, 19–25 g). Monitoring of tumor growth was implemented weekly by means of measuring the tumor width (W) and length (L) with calipers, where the tumor volume (V) was calculated: V = W2 × L)/2. At four weeks post injection, mice were euthanized whereupon the weight of tumor was measured.

### Statistical analysis

Statistical analysis for all data in the current study was implemented by means of SPSS21.0 (IBM Corp. Armonk, NY, USA). The measurement data were displayed as a form of mean ± standard deviation. While following the normal distribution and homogeneity of variance, pair-designed data between two groups were analyzed using paired t-test and unpair-designed data between two groups were analyzed using unpaired t-test. Difference analysis among multiple groups was implemented by means of one-way analysis of variance (ANOVA), followed by Tukey’s post-hoc tests. The data comparison at varied time points was analyzed by means of Bonferroni-corrected repeated-measures ANOVA. Pearson correlation was used to analyze the relationship between two factors. A *p* < 0.05 demonstrated statistical significance.

## Results

### The possible circ_001287/miR-144/CEP55 axis in RCC

**Fig. 1:**
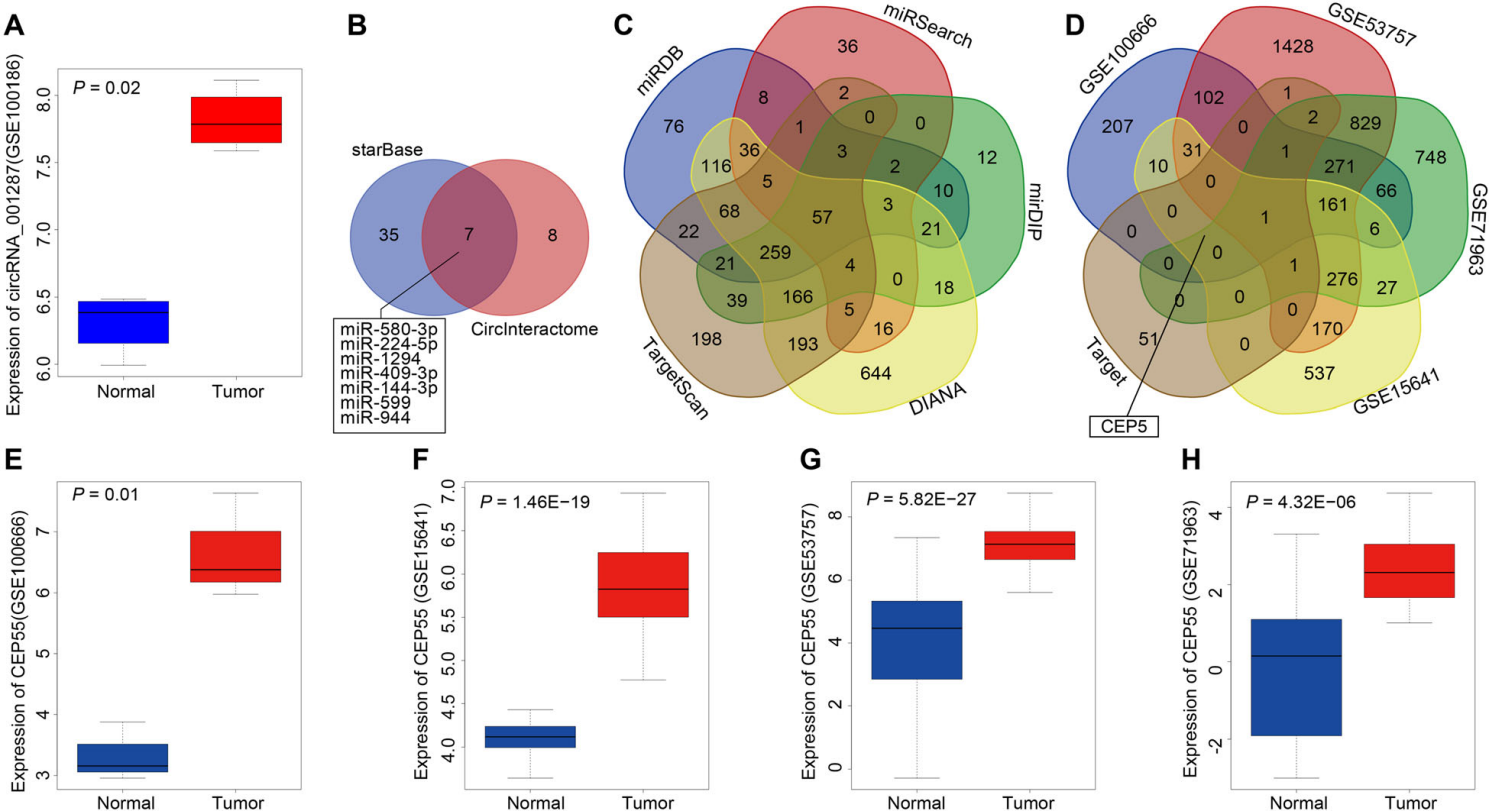
The involvement of circ_001287/miR-144/CEP55 axis in RCC development. **a**, Expression of circ_001287 in RCC-related expression dataset GSE100186. The x-axis represents the sample type, the y-axis represents the expression value, and the upper left stands for the *p* value. **b**, Comparison results of possible miRNA targets for circ_001287 predicted from the CircInteractome and starBase databases. **c**, Comparison results of miR-144 target genes predicted from the DIANA, TargetScan, miRDB, miRSearch and mirDIP databases. **d**, Comparison results of DEGs retrieved from the expression datasets GSE100666, GSE15641, GSE53757, and GSE71963 and target genes of miR-144. **e**, Expression of CEP55 in the expression datasets GSE100666. **f**, Expression of CEP55 in the expression datasets GSE15641. **g**, Expression of CEP55 in the expression datasets GSE53757. **h**, Expression of CEP55 in the expression datasets GSE71963. circRNA, circular RNA; miR-144, microRNA-144; CEP55, centrosomal protein 55

Microarray-based analysis was utilized to identify the RCC-related circRNA and its downstream miRNA as well as the target gene of the downstream miRNA. The differential expression of circRNAs in RCC dataset (GSE100186) showed higher expression of circ_001287 in RCC tissues relative to adjacent normal tissues (Fig. 1a). As found in the circBase, circ_001287 was also referred to as hsa_circ_0000990. The potential miRNA targets for circ_001287 were then predicted by means of CircInteractome and starBase databases with the type of binding sites between circRNA and miRNA defined as 7mer-m8. Forty-two miRNAs were found in starBase database and fifteen in CircInteractome database with context + score percentile > 90. Seven miRNAs were intersected after comparison of the predicted miRNAs (Fig. 1b). Further comparison of their binding sites with circ_001287 showed that the miR-144 and miR-944 had the consistent binding site in two databases. Studies had shown that miR-144 can inhibit the development of RCC [17, 26]. Therefore, miR-144 was selected as a key miRNA for the current research. The target gene of miR-144 was predicted in DIANA, TargetScan, miRDB, miRSearch and miRDIP databases. A total of 708 target genes with target Score > 70 were found in miRDB database and 615 target genes with Integrated Score > 0.7, in miRDIP. Furthermore, 1611, 1043 and 178 target genes were predicted in DIANA, TargetScan and miRSearch databases respectively. The prediction results of target genes were intersected by Venn diagrams and 57 genes were in the intersection (Fig. 1c). The intersected genes were characterized as target genes of miR-144 and compared with the DEGs in the datasets (GSE100666, GSE15641, GSE53757 and GSE71963). The Venn diagram was constructed and we found that only CEP55 was in the intersection (Fig. 1d). Moreover, it was shown that in the GSE100666, GSE15641, GSE53757 and GSE71963 datasets, the expression of CEP55 was higher seen in RCC tissues than that witnessed in adjacent normal tissues (Fig. 1e-h). Taken together, the bioinformatics analysis showed that upregulated circ_001287 and CEP55 in RCC and most importantly, miR-144 seems to involve in the regulation of these two genes.

### circ_001287 is highly expressed in RCC tissues and cells

**Fig. 2:**
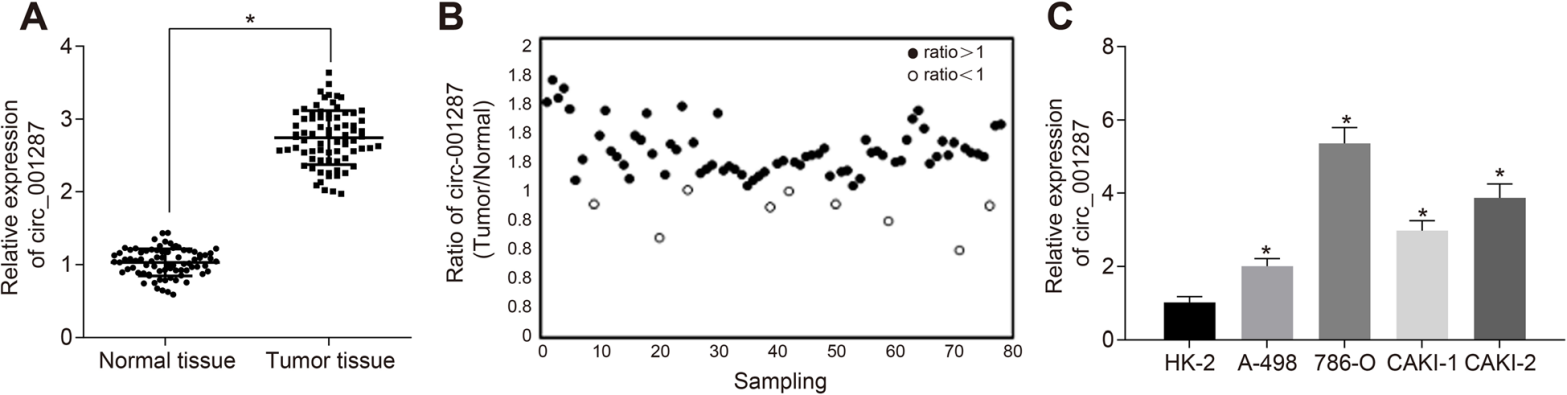
Increased expression of circ_001287 in RCC tissues and cells. **a**, Expression of circ_001287 in RCC tissues and adjacent normal tissues detected using RT-qPCR (n = 77). **b**, Scatter plot of circ_001287 expression in RCC tissues and adjacent normal tissues. **c**, Expression of circ_001287 in normal renal tubular epithelial cell line and RCC cell lines using RT-qPCR. ^*^p < 0.05 vs. normal adjacent tissues or HK-2 cell line. The data were measurement data and presented as mean ± standard deviation. Data between two groups were analyzed using paired t-test and data among groups were compared with one-way ANOVA, followed by Tukey’s post hoc test. The cell experiment was repeated 3 times. circRNA, circular RNA; RT-qPCR, reverse transcription quantitative polymerase chain reaction

Next, the expression of circ_001287 in RCC was explored. Initially, the expression of circ_001287 in RCC tissues and adjacent normal tissues from 77 RCC patients was measured using RT-qPCR. Results showed that expression of circ_001287 was even higher in RCC tissues than that found in adjacent normal tissues (*p* < 0.05; Fig. 2a-b). Based on the mean circ_001287 expression in patients with RCC (2.746), these patients were assigned into high expression group (*n* = 40) and low expression group (*n* = 37). Then, the relationship between the circ_001287 expression and the clinicopathological characteristics of RCC patients was evaluated, which demonstrated that circ_001287 expression was strongly linked to the pathological grade, lymph node involvement, distant metastasis, tumor size, and tumor node metastasis (TNM) stage of RCC patients. However, no correlation was noted between the circ_001287 expression and the age, gender, as well as expression of UACR, eGFR, Cr, and CysC of RCC patients. These results are listed in Table 1. Further, expression of circ_001287 in normal renal tubular epithelial cell line and RCC cell lines was detected using RT-qPCR. We found that the expression of circ_001287 in these renal cell lines A-498, 786-O, CAKI-1 and CAKI-2 was higher than that in normal renal tubular epithelial HK-2 cell line and the 786-O cell line exhibited the highest expression of circ_001287 (all *p* < 0.05; Fig. 2c). Therefore, 786-O was selected for subsequent experiments. These results reveal that circ_001287 is upregulated in RCC tissues and cells.

### Depletion of circ_001287 suppresses malignant phenotype of RCC cells

**Fig. 3:**
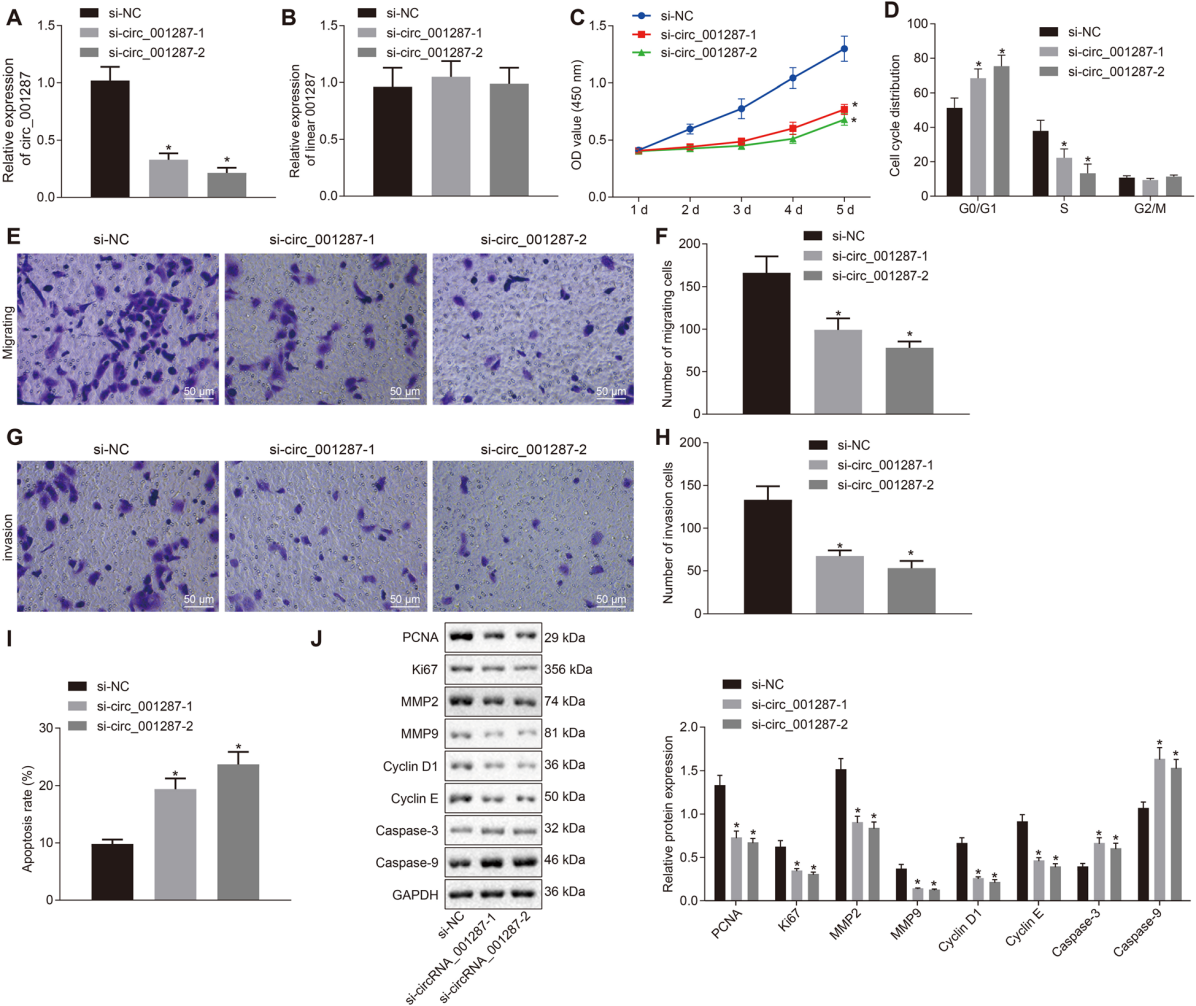
Down-regulated circ_001287 contributes to repressed proliferation, migration and invasion abilities in RCC cells. The 786-O cells were transfected with si-NC, si-circ_001287-1, or si-circ_001287-2. **a**, Expression of circ_001287 in 786-O cells measured using RT-qPCR. **b**, Expression of linear RNA_001287 in 786-O cells detected using RT-qPCR. **c**, Viability of 786-O cells measured using CCK-8 assay. **d**, Cell cycle distribution of 786-O cells determined using flow cytometry. **e**, Image (× 200) of migration of 786-O cells measured using Transwell assay. **f**, Quantification analysis of migration ability of 786-O cells measured using Transwell assay. **g**, Image (× 200) of invasion of 786-O cells evaluated using Transwell assay. **h**, Quantification analysis of invasion ability of 786-O cells evaluated using Transwell assay. **i**, The 786-O cell apoptosis assessed using flow cytometry. **j**, PCNA, KI67, MMP2, MMP9, Cyclin D1, Cyclin E, Caspase-3, and Caspase-9 protein expression in 786-O cells measured using western blot analysis. ^*^p < 0.05 vs. 786-O cells treated with si-NC. The data were measurement data and described as mean ± standard deviation. Data among multiple groups were compared with one-way ANOVA, followed by Tukey’s post hoc test. The cell experiment was repeated 3 times. circRNA, circular RNA; RT-qPCR, reverse transcription quantitative polymerase chain reaction; CCK-8, cell counting kit-8

After determining the expression of circ_001287 in RCC, we further explored whether circ_001287 has an impact on the biological function of RCC. We constructed two siRNAs: si-circ_001287-1 and si-circ_001287-2 and transfected them into cells. First, in order to identify whether the transfected siRNA sequence specifically decreased the expression of circ_001287, relative expression of circ_001287 and linear RNA_001287 was measured respectively using RT-qPCR. Circ_001287 relative expression after treatment with si-circ_001287-1 and si-circ_001287-2 was reduced, and linear RNA_001287 expression did not differ obviously (Fig. 3a-b), indicating the successful silencing efficiency. Next, CCK-8 assay showed that number of cells in each group increased over time. The 786-O viability was decreased by si-circ_001287-1 or si-circ_001287-2 (*p* < 0.05; Fig. 3c). Flow cytometry and Transwell assay displayed that after si-circ_001287-1 or si-circ_001287-2 treatment, 786-O cells arrested in G0/G1 phase were increased, accompanied by decline of cells in S phase (Fig. 3d), invasive and migratory capacities were diminished (Fig. 3e-h), and cell apoptosis was elevated (Fig. 3i) (all *p* < 0.05). Furthermore, the expression of proliferation- (PCNA and KI67), migration and invasion- (MMP2 and MMP9), cell cycle- (Cyclin D1 and Cyclin E) and apoptosis-related proteins (Caspase-3 and Caspase-9) was measured using western blot analysis. It was demonstrated that protein expression of PCNA, KI67, MMP2, MMP9, Cyclin D1 and Cyclin E was down-regulated, while that of Caspase-3 and Caspase-9 was up-regulated in cells treated with si-circ_001287 versus cells treated with si-NC (all *p* < 0.05; Fig. 3j).

The above findings indicate that the down-regulation of circ_001287 inhibits the proliferative, invasive and migratory capacities and promotes the apoptosis of RCC cells.

### circ_001287 specifically binds to miR-144

**Fig. 4:**
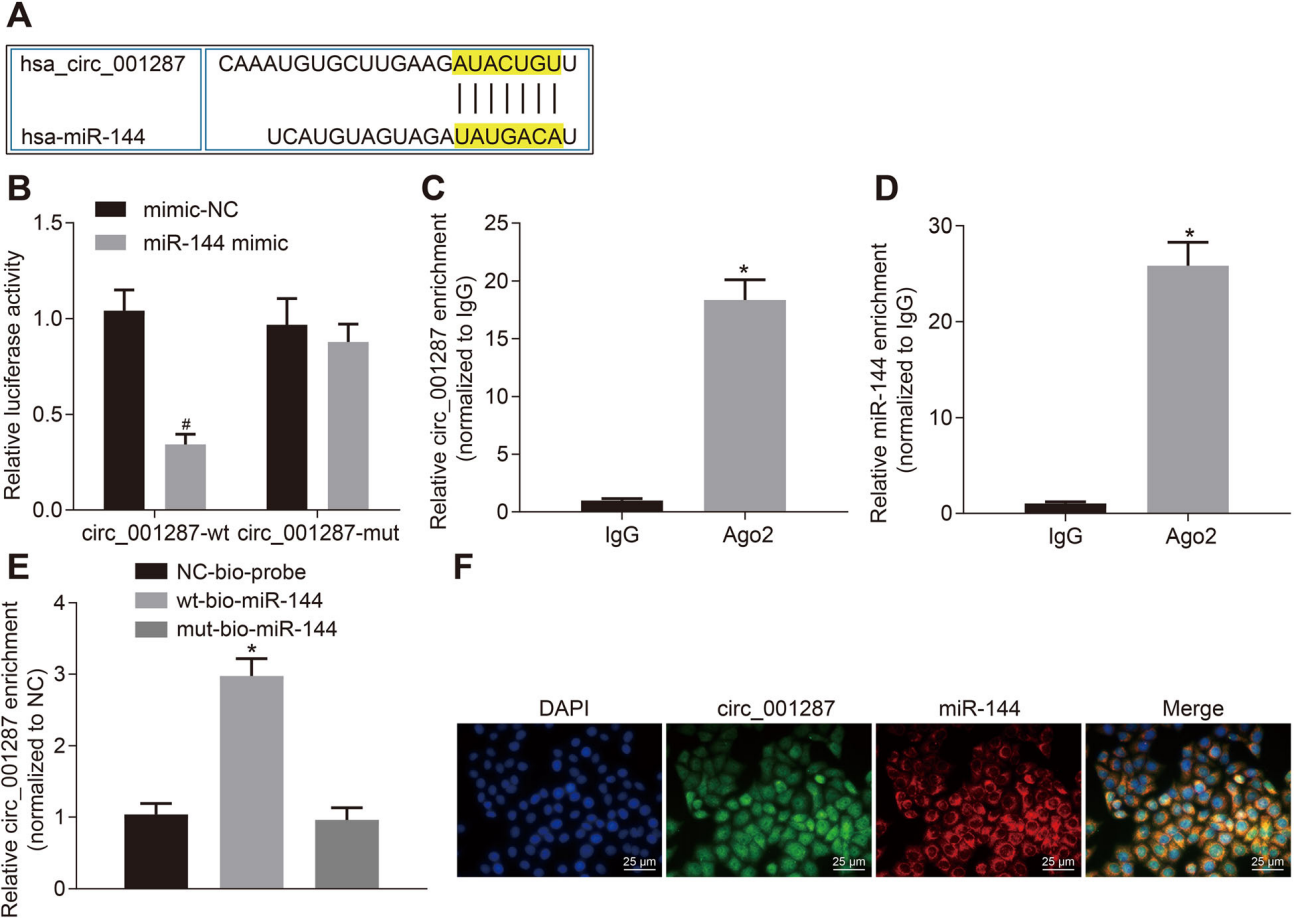
miR-144 can specifically bind to circ_001287. **a**, Binding sites between circ_001287 and miR-144 predicted through a bioinformatics website. **b**, Binding relationship of miR-144 to circ_001287 measured using dual-luciferase reporter gene assay. **c**, Binding of circ_001287 and Ago2 measured using RIP assay. **d**, Binding of miR-144 and Ago2 measured using RIP assay. **e**, Binding of circ_001287 and miR-144 analyzed using RNA pull-down assay. **f**, Co-location of circ_001287 and miR-144 in the cytoplasm tested using FISH (× 400). # *p* < 0.05 vs. the cells treated with mimic-NC; ^*^p < 0.05 vs. IgG and NC-bio-probe. Statistical data were measurement data and described as mean ± standard deviation. Independent sample t-test was used for data comparison between the two groups. Data among groups were compared with one-way ANOVA together with Tukey’s post hoc test. The cell experiment was repeated 3 times. circRNA, circular RNA; miR-144, microRNA-144; Ago2, Argonaut-2; RIP, RNA immunoprecipitation; FISH, Fluorescence in situ hybridization

To understand whether circ_001287 interacts with miR-144, the bioinformatics website (https://circinteractome.nia.nih.gov/index.html) was used to explore the potential miRNA regulated by circ_001287 and our analysis indicated that there was a potential binding site between circ_001287 and miR-144 (Fig. 4a). Dual-luciferase reporter gene assay was implemented to verify this assumption. As expected, the luciferase activity of circ_001287-wt was attenuated in response to miR-144 mimic. However, the luciferase activity of circ_001287-mut remained unchanged (Fig. 4b). Meanwhile, RIP assay documented that compared with IgG, circ_001287 and miR-144 binding to Ago2 were increased (*p* < 0.05; Fig. 4c-d), indicating that both circ_001287 and miR-144 can bind to Ago2. Furthermore, RNA-pull down assay described that compared with mut-miR-144, wt-miR-144 bound to more Ago (*p* < 0.05; Fig. 4e). Additionally, FISH analysis manifested that both circ_001287 and miR-144 were located in the cytoplasm of RCC cells (Fig. 4f). Collectively, circ_001287 can directly bind to miR-144.

### miR-144 down-regulation promotes the malignant biological behaviors of RCC cells to reverse the effect of circ_001287 silencing

**Fig. 5:**
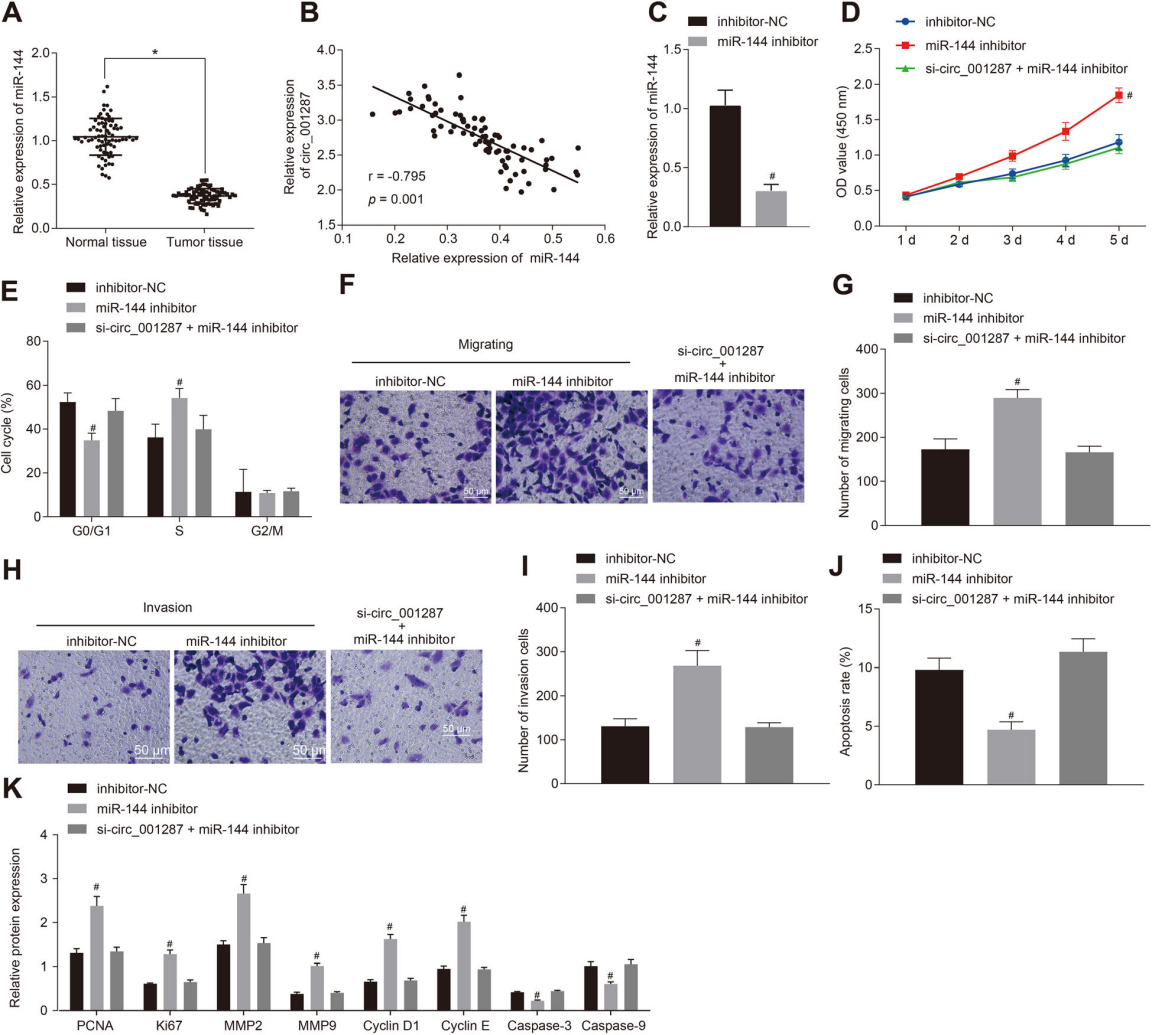
miR-144 down-regulation promotes proliferation, migration and invasion and inhibits apoptosis in RCC cells. **a**, Expression of miR-144 in RCC tissues and adjacent normal tissues measured using RT-qPCR (*n* = 77). **b**, Correlation analysis between circ_001287 and miR-144. **c**, Relative expression of miR-144 in 786-O cells after treatment with miR-144 inhibitor using RT-qPCR. The 786-O cells were transfected with miR-144 inhibitor, inhibitor NC, or si-circ_001287 + miR-144 inhibitor. **d**, Viability of 786-O cells measured using CCK-8 assay. **e**, Cell cycle distribution measured using flow cytometry. **f**, Image (× 200) of migration of 786-O cells measured using Transwell assay. **g**, Quantification analysis of migration ability of 786-O cells measured using Transwell assay. **h**, Image (× 200) of invasion of 786-O cells evaluated using Transwell assay. **i**, Quantification analysis of invasion ability of 786-O cells evaluated using Transwell assay. **j**, Apoptosis in 786-O cell assessed through flow cytometry. **k**, PCNA, KI67, MMP2, MMP9, Cyclin D1, Cyclin E, Caspase-3, and Caspase-9 protein expression in 786-O cells measured using Western blot analysis. ^*^p < 0.05 vs. adjacent normal tissues or 786-O cells treated with inhibitor NC; # *p* < 0.05 vs. the 786-O cells treated with miR-144 inhibitor. The data were measurement data and described as mean ± standard deviation. Paired t-test was used for data comparison between the two groups. Data among groups were compared with one-way ANOVA along with Tukey’s post hoc test. The data at different time points among different groups was analyzed by the repeated-measures ANOVA and Bonferroni’s post hoc test. The cell experiment was repeated 3 times. miR-144, microRNA-144; RT-qPCR, reverse transcription quantitative polymerase chain reaction; circRNA, circular RNA; CCK-8, cell counting kit-8

To dissect out the effects of miR-144 on the biological function in RCC, RT-qPCR exhibited miR-144 expression was even lower in RCC tissues than that seen in adjacent normal tissues (Fig. 5a), and circ_001287 expression was inversely correlated with the miR-144 expression (Fig. 5b). After the 786-O cells were transfected with miR-144 inhibitor, miR-144 expression was markedly reduced, suggesting the successful transfection efficiency (*p* < 0.05, Fig. 5c). Cell viability (Fig. 5d), cell cycle (Fig. 5e), the migration and invasion (Fig. 5f-i) and cell apoptosis (Fig. 5j) were determined. Results showed that 786-O cell viability, migration and invasion after miR-144 inhibitor treatment were significantly accelerated, whereas the apoptotic rate was lowered, accompanied by reduced cells in the G0/G1 phase and increased cells in S phase, which was annulled by the further treatment of si-circ_001287 in 786-O cells (all *p* < 0.05).

Additionally, Western blot analysis exhibited that protein expression pattern of PCNA, KI67, MMP2, MMP9, Cyclin D1 and Cyclin E was facilitated and that of Caspase-3 and Caspase-9 was lowered by miR-144 inhibitor, which was abrogated by additional silencing circ_001287 (*p* < 0.05; Fig. 5k).

In summary, inhibition of miR-144 promoted the malignant biological behaviors of RCC cells, which normalized the effect of silencing circ_001287.

### circ_001287 orchestrates miR-144-targeted CEP55 to suppress the malignant biological behaviors of RCC cells

**Fig. 6:**
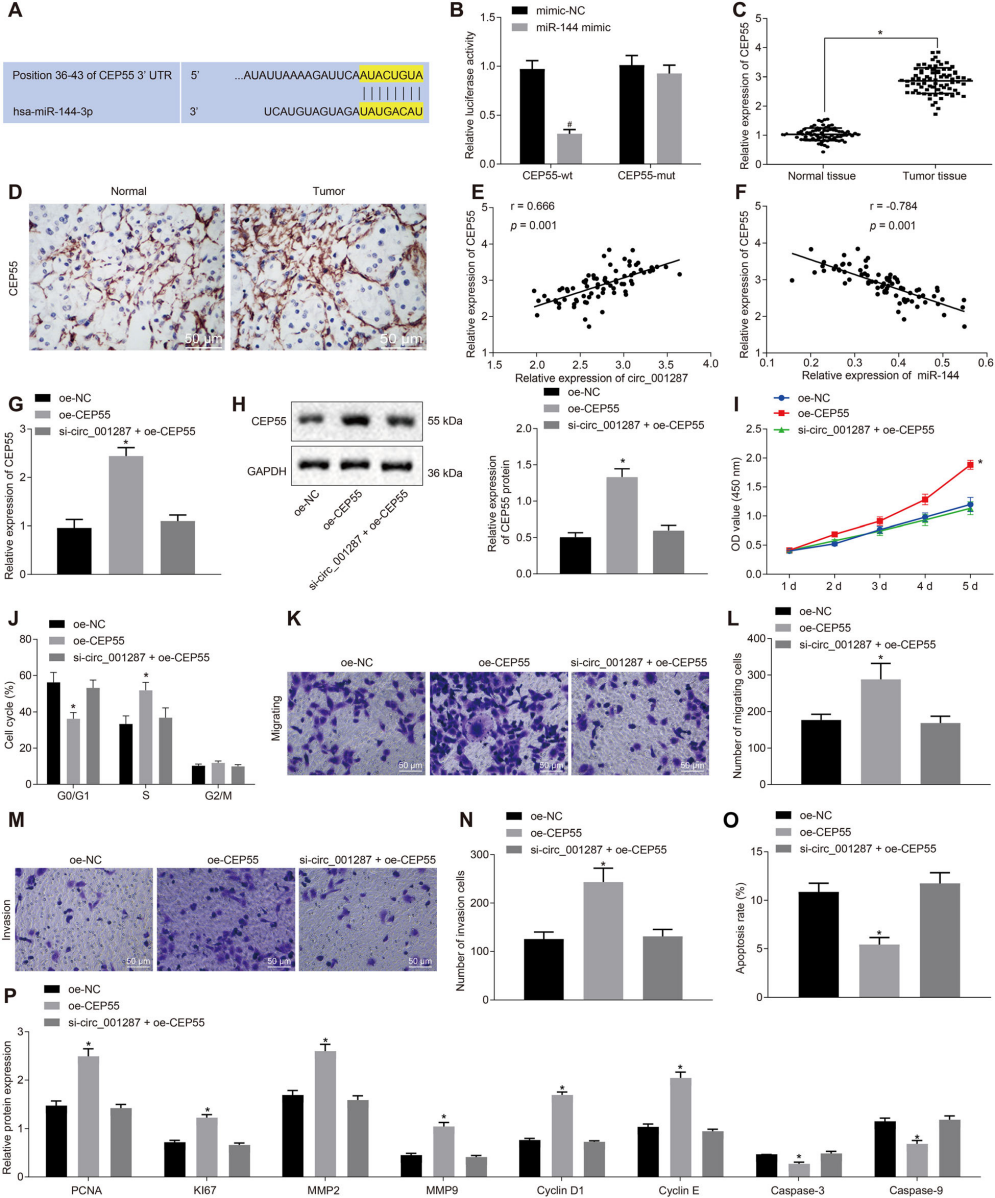
circ_001287 modulates miR-144-targeted CEP55 and thus hinders the progression of RCC. **a**, Binding sites between miR-144 and CEP55 predicted through a bioinformatics website. **b**, Targeting relationship between miR-144 and CEP55 tested by dual-luciferase reporter gene assay. **c**, Expression of CEP55 in RCC tissues and adjacent normal tissues measured using RT-qPCR (*n* = 77). **d**, Expression of CEP55 in RCC tissues and adjacent normal tissues measured using immunohistochemistry (× 200). **e**, Correlation analysis of circ_001287 and CEP55. **f**, Correlation analysis of miR-144 and CEP55. The 786-O cells were treated with oe-NC, oe-CEP55, or si-circ_001287 + oe-CEP55. **g**, mRNA expression of CEP55 in 786-O cells determined using RT-qPCR. **h**, Protein expression of CEP55 in 786-O cells tested using western blot analysis. **i**, Viability of 786-O cells measured using CCK-8 assay. **j**, Cell cycle distribution measured using flow cytometry. **k**, Image (× 200) of migration of 786-O cells measured using Transwell assay. **l**, Quantification analysis of migration ability of 786-O cells measured using Transwell assay. **m**, Image (× 200) of invasion of 786-O cells evaluated using Transwell assay. **n**, Quantification analysis of invasion ability of 786-O cells evaluated using Transwell assay. **o**. Apoptotic rate in 786-O cell analyzed using flow cytometry. **p**, PCNA, KI67, MMP2, MMP9, Cyclin D1, Cyclin E, Caspase-3, and Caspase-9 protein expression in 786-O cells measured using western blot analysis. ^*^p < 0.05 vs. adjacent normal tissues or 786-O cells treated with oe-NC; # *p* < 0.05 vs. the 786-O cells treated with oe-CEP55. The data were measurement data and described as mean ± standard deviation. Paired t-test was used for data comparison between RCC tissues and adjacent normal tissues. Unpaired t-test was used for analysis of data between the other two groups. Data among groups were compared with one-way ANOVA, followed by Tukey’s post hoc test. The data at different time points among different groups were analyzed by the repeated-measures ANOVA and Bonferroni’s post hoc test. The cell experiment was repeated 3 times. miR-144, microRNA-144; CEP55, centrosomal protein 55; RT-qPCR, reverse transcription quantitative polymerase chain reaction; circRNA, circular RNA; mRNA, messenger RNA; CCK-8, cell counting kit-8

Given that miR-144 acted an important role in the development of RCC, it is essential to find out the downstream mechanism of miR-144 in RCC. The website (http://www.targetscan.org/vert_71/) predicted a binding site existed between miR-144 and CEP55 (Fig. 6a). Meanwhile, the luciferase activity of CEP55-3’UTR-wt was inhibited by miR-144 mimic (*p* < 0.05). However, the luciferase activity of CEP55-3’UTR-mut was not affected (*p* > 0.05; Fig. 6b), suggesting that miR-144 can specifically bind to 3’UTR of CEP55 and down-regulate CEP55. Following this, CEP55 expression was higher in RCC tissues than in adjacent normal tissues, as measured by RT-qPCR and immunohistochemistry (Fig. 6c-d). Moreover, circ_001287 expression was positively correlated with CEP55 expression (Fig. 6e). On the other hand, an inverse correlation was observed between miR-144 and CEP55 expression (Fig. 6f). RT-qPCR and Western blot analysis illustrated that CEP55 expression was elevated in oe-CEP55-treated 786-O cells, which was neutralized by further si-circ_001287 treatment (*p* < 0.05; Fig. 6g-h).

Subsequently, results showed that CEP55 overexpression resulted in enhancement of viability (Fig. 6i), decline of cells in the G0/G1 phase (Fig. 6j), increase of migration and invasion (Fig. 6k-n), and reduction of apoptotic rate (Fig. 6o) of 786-O cells, whilst further circ_001287 silencing abrogated these trends. As reflected by western blot analysis, up-regulated PCNA, KI67, MMP2, MMP9, Cyclin D1 and Cyclin E and down-regulated Caspase-3 and Caspase-9 were observed in CEP55-overexpressed 786-O cells, which was counteracted by silencing circ_001287 (all *p* < 0.05; Fig. 6p).

Taken together, circ_001287 increases CEP55 expression and further depresses the malignant biological behaviors of RCC cells.

### Down-regulated circ_001287 augments miR-144 expression and then diminishes CEP55, leading to the attenuation of xenograft tumorigenesis in nude mice

**Fig. 7:**
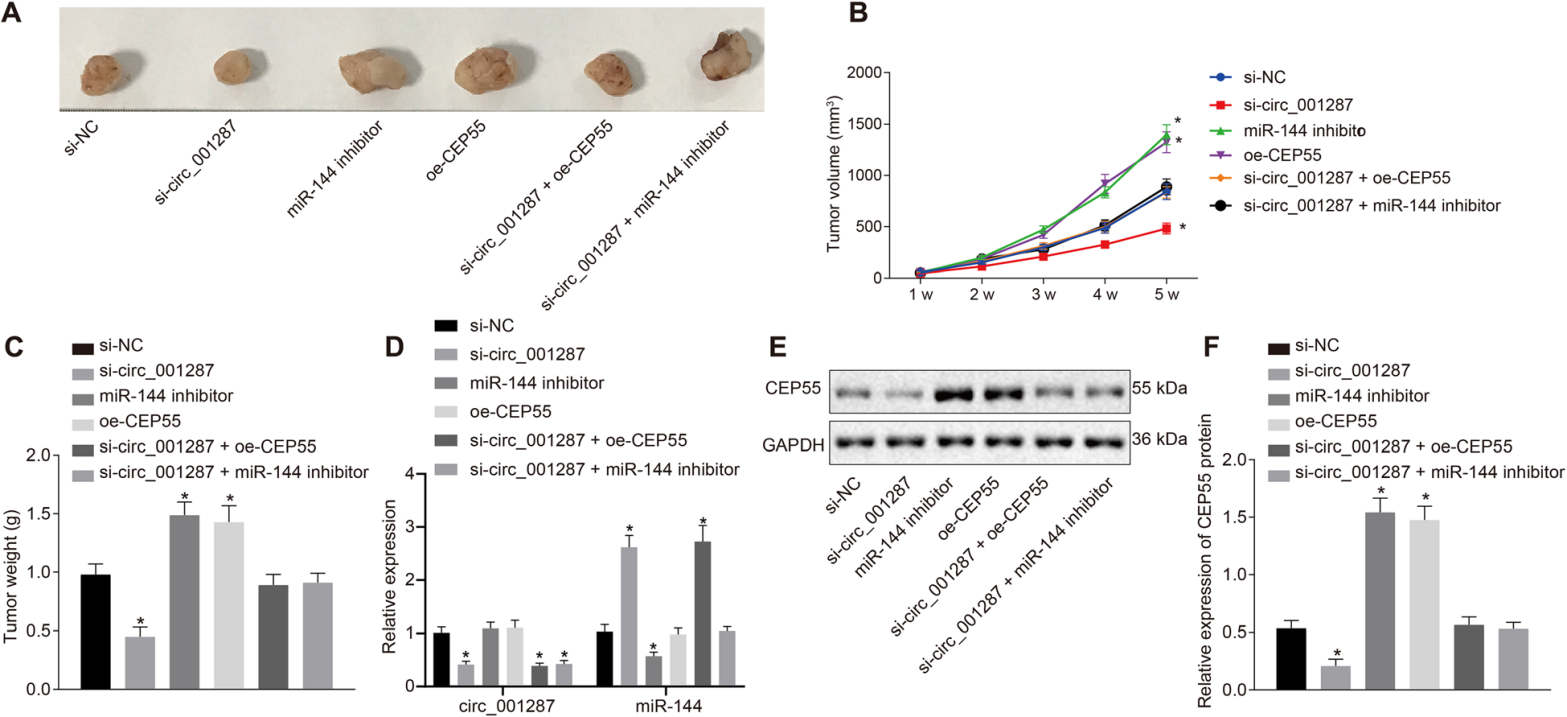
Depleted circ_001287 reduces miR-144-mediated CEP55 expression to inhibit xenograft tumorigenesis in nude mice. Nude mice were injected with 786-O cells transfected with si-NC, si-circ_001287, miR-144 inhibitor, oe-CEP55, si-circ_001287 + miR-144 inhibitor, or si-circ_001287 + oe-CEP55. **a**, Representative images of xenograft tumors in nude mice. **b**, Tumor volume in mice. **c**, Tumor weight in mice. **d**, The expression of circRNA-001287 and miR-144 in tumor samples determined with RT-qPCR. **e**, Gray value of CEP55 protein bands in mice evaluated by western blot analysis. **f**, Protein expression of CEP55 in nude mice measured by western blot analysis. ^*^p < 0.05 vs. mice injected with si-NC-transfected 786-O cells. Statistical data were measurement data and described as mean ± standard deviation. Data among multiple groups were compared with one-way ANOVA, along with Tukey’s post hoc test. The data at different time points among different groups were analyzed by the repeated-measures ANOVA and Bonferroni’s post hoc test (n = 10). CEP55, centrosomal protein 55

The above results have revealed the roles of circ_001287 in RCC *in vitro* and herein, we further investigated the effect of circ_001287 on xenograft tumorigenesis in nude mice. The tumor volume was measured after injection. The results (Fig. 7a-c) manifested that the volume of the tumors increased gradually with time. Meanwhile, the average volume and weight of tumor were reduced in mice following injection with si-circ_001287-treated cells (*p* < 0.05). Conversely, the average volume and weight of tumor were elevated in mice injected with miR-144 inhibitor-treated cells or with oe-CEP55-treated cells, which was negated by additional treatment with si-circ_001287 (*p* < 0.05). RT-qPCR demonstrated that si-circ_001287 induced reduction in circ_001287 expression and potent rise in miR-144 expression. miR-144 expression was lowered in response to miR-144 inhibitor and circ_001287 expression remained unchanged after treatment with miR-144 inhibitor or oe-CEP55. In addition, the mice co-treated with si-circ_001287 and oe-CEP55 exhibited reduced circ_001287 expression as well as elevated miR-144 expression in contrast to the si-NC treatment. circ_001287 expression in mice was diminished in response to si-circ_001287 + miR-144 inhibitor, while miR-144 expression remained unchanged compared to si-NC treatment (Fig. 7d). Moreover, western blot analysis exhibited decline in CEP55 protein expression in response to si-circ_001287 (*p* < 0.05). Treatment with miR-144 inhibitor or oe-CEP55 led to elevated CEP55 protein expression, which was annulled by additional treatment with si-circ_001287 (*p* < 0.05; Fig. 7e, f) The results suggest that downregulation of circ_001287 can decrease CEP55 expression by enhancing miR-144 expression, thus inhibiting the tumorigenicity of RCC cells *in vivo*.

## Discussion

Surgery is an option for patients with early stage of localized RCC while unfortunately, localized disease has the potential to undergo early hematogenous dissemination, thus leading to metastasis [27]. Previous studies have reported the functionality of circRNAs in the pathogenesis of various human diseases such as cardiovascular diseases, neurological diseases, osteoarthritis, diabetes, and especially urological cancers [28]. However, only few data focus on the association between circRNAs and RCC pathogenesis. In this study, we aim to define the possible role of circ_001287 in RCC. Both *in vitro* and *in vivo* experimental results demonstrated that circ_001287 can stimulate proliferative, invasive and migratory capacities while delay apoptosis of RCC cells by binding to miR-144 and upregulation of CEP55.

Initially, our data showed a significant upregulated expression of circ_001287 in both RCC tissues and cell lines when compared with normal controls. The widespread distribution of circRNAs in human cells has been established, with much higher expression than that of linear isomers [29]. A lot of evidence supports the increased expression of circRNAs in RCC. circPCNXL2 has been found to be significantly upregulated in RCC cells and correlates with poor overall survival in these patients [30]. In addition, the expression of circ-ZNF609 has been observed to be increased in RCC and upregulation of this circRNA promotes cell proliferative and invasive capacities [31]. These observations were in agreement with our findings that circ_001287 was able to drive RCC cell proliferative and invasive capacities while impeding cell apoptosis.

Another key important observation was that circ_001287 can bind to miR-144 and downregulate its expression. Similarly, hsa_circ_0020123 has been recognized to competitively bind with miR-144 and then exerts oncogenic properties in the context of non-small cell lung cancer [32]. Recent study suggests that miR-144 may play a key role in tumorigenesis and cancer therapy, and functions of miR-144 are tissue-specific [33]. In our current study, miR-144 expression was shown to be dramatically decreased in RCC tissues and cell lines. Consistent with our results, miR-144-3p expression is even lower in RCC specimens and cell lines. Additionally, upregulation of miR-144-3p inhibits RCC cell proliferation and progression *in vitro* [26].

Some circRNAs can regulate miRNAs to function and the circRNA-miRNA-mRNA axis demonstrates crucial effects in the context of cancer-related or non-cancer pathways [34]. For instance, circ-ZNF609 works as a ceRNA to control FOXP4 expression by means of binding to miR-138-5p in renal carcinoma [31]. Moreover, mechanistic investigations suggest that hsa_circ_0008039 serves as a ceRNA of miR-432-5p and elevated E2F3 that is identified as a functional target of miR-432-5p, which significantly suppress the proliferation, arrest cell-cycle progression and reduce migration of breast cancer cells [35]. In our work, bioinformatics prediction combined with luciferase reporter assay verified CEP55 being a direct target gene of miR-144 which had the potency to cease its expression. Consistently, miR-144 targets and negatively regulates CEP55 in breast cancer [18]. Furthermore, Li et al.. find that circPCNXL2 binds to miR-153 to promote the proliferative and invasive capacities of RCC cells through upregulating ZEB2 [30]. These results supported our conclusion that circ_001287 upregulation can increase CEP55 expression by means of competitive binding to miR-144, thereby accelerating the proliferative, invasive and migratory capacities of RCC cells while decelerating apoptosis.

## Conclusions

In summary, our study reveals the property of the circ_001287/miR-144/CEP55 regulatory network in RCC in which overexpressed circ_001287 potentially exacerbates the progression of RCC through miR-144 suppression and CEP55 elevation. Our findings provide a new perspective for the pathogenesis of RCC. In the future, the molecular mechanism of circ_001287 in RCC still requires additional work in a more detailed manner with different cell lines.

## Data Availability

The datasets used and/or analyzed in the current study are attained from the corresponding author on reasonable request.

## Abbreviations

RCC: Renal cell carcinoma
circRNAs: Circular RNAs
CEP55: Centrosomal protein 55
EMT: Epithelial-mesenchymal transition
FBS: Fetal bovine serum
FISH: Fluorescence in situhybridization
RT-qPCR: Reverse transcription quantitative polymerase chain reaction
WT: Wild type
RIP: RNA binding protein immunoprecipitation
IgG: Immunoglobulin G
MMP2: Matrix metalloproteinase 2
CCK-8: Cell counting kit-8
